# BRIDGING THE DIGITAL DIVIDE: A USER-CENTERED DESIGN APPROACH TO ENHANCING SOCIAL CONNECTIVITY AMONG OLDER ADULTS

**DOI:** 10.1093/geroni/igae098.4135

**Published:** 2024-12-31

**Authors:** Edward Chanquin, Sheila Salinas Navarro, Francesca Falzarano

**Affiliations:** University of Southern California, Los Angeles, California, United States; University of Southern California, Los Angeles, California, United States; University of Southern California, Los Angeles, California, United States

## Abstract

Social isolation is highly prevalent among older adults, leading to negative health outcomes and increased healthcare costs. With the U.S. Census Bureau projecting a near doubling of adults aged 65 and older by 2060, addressing this issue is crucial. Innovative, user-centered solutions are imperative because older adults often face health, cognitive, and mobility limitations that diminish opportunities for engagement. Technologies can help overcome these barriers. This study aimed to develop a potential intervention to enhance social connectivity among older adults through user-centered design, understanding their needs in using digital technologies. This qualitative study employed an ethnographic approach, engaging 50 older adults in Los Angeles. Semi-structured interviews and participant observations were conducted. Thematic analysis informed the iterative design of a prototype named TwinFlame. The study revealed three key insights: 1. Older adults prioritized user-friendly interfaces facilitating communication with loved ones. 2. Participants expressed a need for educational support in using digital devices, particularly for advanced features. 3. Cultural competence was critical in ensuring inclusivity and acceptance of the proposed solution. TwinFlame, a device resembling a candle with integrated digital communication features, was developed to address these needs. Its design incorporates simplified interfaces, built-in tutorials, and culturally sensitive elements. While not yet tested, TwinFlame’s potential for enhancing social connectivity lies in its user-centered approach, directly addressing the identified needs of older adults. This research contributes to understanding how tailored digital tools can potentially reduce the digital divide and social isolation among older adults, highlighting the importance of user-centered design in gerontechnology.

